# Method for fabricating self-powered cement sensors based on gold nanoparticles

**DOI:** 10.1016/j.mex.2023.102280

**Published:** 2023-07-04

**Authors:** Daniel A. Triana-Camacho, Rogelio Ospina-Ospina, Jorge H. Quintero-Orozco

**Affiliations:** Escuela de Física, Universidad Industrial de Santander, Cra 27 Calle 9, Bucaramanga, Colombia

**Keywords:** Cement-based composites, Gold nanoparticles, Piezoelectricity, Pulsed laser ablation in liquid, *Piezoelectric properties for cement-based gold nanoparticles*

## Abstract

Nowadays, cement industry researchers are working hard to develop cement sensors based on nanocomposites because they can be used to develop intelligent and sustainable civil structures, self-powered, self-healing, or self-monitoring. In this light, this paper shows a methodology to obtain piezoelectric cement sensors, which produce enough energy not to require an external power source in sensing-strain applications. Mainly, two proposed experimental procedures increased the piezoelectric properties of these cement-based composites: add gold nanoparticles in the proper concentrations and apply a constant electric field during the curing stage. Firstly, the gold nanoparticles were obtained through a pulsed laser ablation system, and their particle size distribution was measured with a particle analyzer Litesizer 500 from Anton Paar, and their morphology was corroborated using a scanning electron microscope. Two concentrations (442 ppm and 658 ppm) of gold nanoparticles were obtained by changing the total ablation time. Next, we fabricated the cement sensors as described by ASTM standards C39-C39M. Hence, the cement was hand mixed with a water-to-cement ratio (w/c) of 0.47 for then poured on cylindrical molds saving the proportions recommended by the ASTM standard; in this stage, the gold nanoparticles were already part of the water ratio. Then, the cement sensors were cured under an external electric field and dried for 24 hours more in an oven to be finally ready for electromechanical characterization. Meanwhile, the electric response in altern current and the piezoelectric behavior were corroborated through electrical impedance spectroscopy and open circuit potential measurements, respectively. The piezoelectric behavior was obtained when a compressive strength was applied to the sensor, and the generated voltage was simultaneously measured. Finally, the electrical and mechanical characterization measurements were processed and analyzed using Python scripts.

• The particle size and the families amount of Au NPs are affected by the ablation time.
• The correct proportion of Au NPs increases the inherent piezoelectricity of cement paste.
• The piezoelectric response can be addressed by coupling electric and mechanical tests.

## Graphical abstract

**Figure:**
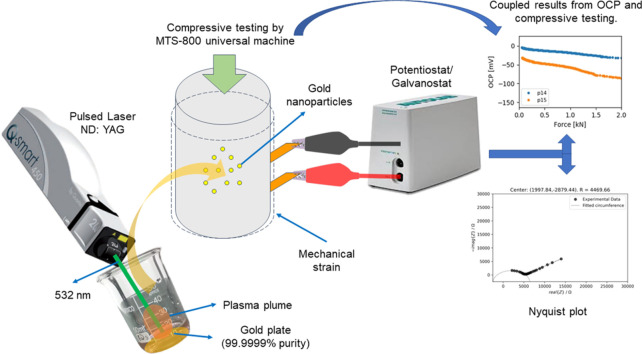

Specifications table

**Table:** 

Subject Area:	Engineering
More specific subject area:	*Cement-based composites*
Method name:	*Piezoelectric properties for cement-based gold nanoparticles*
Name and reference of the original method:	*N.A.*
Resource availability:	*link to software repository:* https://github.com/dantrica/Au-NPs-cement-composites

## Method details

The materials used in this work were a 30 mm diameter and 0.15 mm thickness target gold plate (99.9999% purity), ultra-pure water from a Milli-Q IQ 7000 equipment (18.2 MΩ), general use Portland Cement from ARGOS Colombia, and 2.5 mm copper desoldering wire. The high cost of gold might seem an impediment to fabricating cement-based composites. Also, this could be the main reason why there are no related investigations in that the gold nanoparticles (Au NPs) were added to the cement matrix. Nonetheless, a previous work by Pusty et al. [1] showed that Au NPs added to cellulose significantly increased the piezoelectric response of the new composite. For this reason, in this work, it has been proposed to use Au NPs in very low concentrations to be incorporated into the cement pastes and thus manufacture cement sensors that can be applied in structural health monitoring applications, which generate their own energy and do not depend on input signals to provide any electrical response.

## Gold nanoparticles synthesis and characterization

**Fig. 1:**
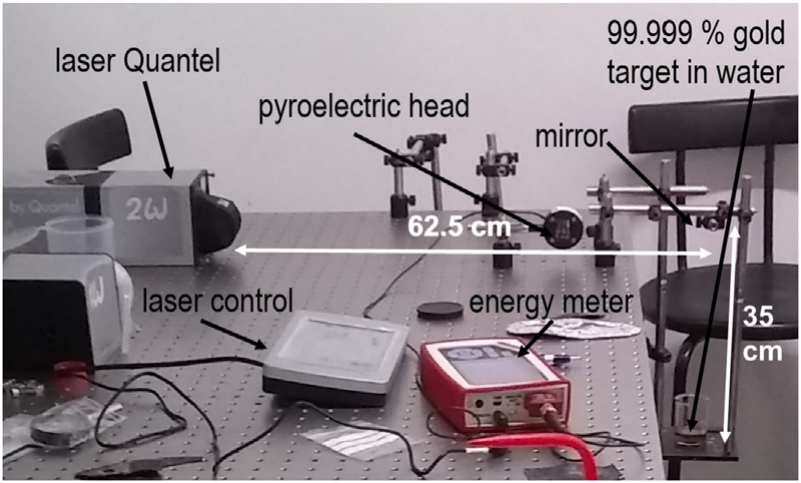
Pulsed laser ablation in liquid system.

A Pulsed Laser Ablation System was used to obtain the Au NPs in ultrapure water as reported reference [2]. This system is composed of (a) a pulsed Nd:YAG laser from Quantel Q-smart 850 with harmonic 2ω (532 nm), which is controlled by the Q-Touch pad; (b) an energy meter console c-series (the energy in Joul has been sensed with the pyroelectric sensor during 5 s to avoid damages on the sensor); (c) bases and post holders (optical mount); and (d) an NB1-K12 mirror to divert the laser ray by 90° and pointed the beaker, which contained (e) a metallic target (gold) immersed in Milli-Q type-1 water, as is presented in Fig. 1.

The setup for obtaining the desired concentration of Au NPs and their particle size is the following: A gold target immersed in Milli-Q type water was exposed to the pulsed laser at 25 °C, the laser wavelength was 532 nm with its laser spot of 12.6 mm^2^, the time between pulses 10^−1^ s, pulse duration 8 ns, and pulse energy 350 mJ. What is more, the water volume and the ablation time are varied to obtain different concentrations in parts per million (ppm) of Au NPs, low 442 ppm, and high 658 ppm. Therefore, low and high concentrations were obtained with the following setups: (water: 10 ml, ablation time: 5 min) and (water: 50 ml, ablation time: 10 min), respectively. In addition, The mass of the gold target it had been measured five times in precision balance Ohaus with a readability of 1.0 μg before and after the ablation stage to calculate the Au NPs concentration in ppm. Nonetheless, two factors three level experimental design was performed to get the desired mean particle size of Au NPs. In that sense, the water volume of 5 mL, 10 mL, and 20 mL; and the laser energy of 250 mJ, 300 mJ, and 350 mJ were varied to find a mean particle size of Au NPs approximately between 100 nm to 500 nm, as was measured in the experimental campaign and will be presented below in this section. Having stated this, why is this particle size range an optimal condition to elaborate self-powered Au NPs-cement sensors? The reasons are: (i) the mechanisms of electric charge conduction between adjacent nanoparticles inside the cement matrix should not have quantum components [3] if we want to interpret the electric or piezoelectric response through classical electrodynamics, i.e., it is necessary to obtain nanoparticle sizes larger than 100 nm where the quantum effects begin to be negligible [4]. Moreover, (ii) the Au NPs should not be large enough to embed in the walls of the micropores and weaken the cement matrix. For instance, Wang et al. [5] reported that those families of micropores have sizes between 5 nm and 360 nm, while Triana-Camacho et al. [6] have obtained families of pores via an effective medium model for electrical properties in alternate current with sizes between 30 nm and 159 nm.

To corroborate the nanometric particles' presence and avoid the agglomeration effects in the physicochemical characterization, the particle size distribution was taken 10 minutes after the physical synthesis of Au NPs, using the dynamic light scattering (DLS) technique in a particle analyzer Litesizer 500 from Anton Paar. To carry out this, the dispersion of Au NPs was tipped out in 1 mm optical path quartz cuvettes without diluting them. Then, the particle size distribution of Au NPs was measured at a 90° angle. Each specimen was tested in triplicate, and the particle analyzer made an average of 6 series of measurements by each test. The results confirmed that a longer ablation time (10 minutes) produces a higher concentration of Au NPs and average particle size d_50_ of 77 ± 4 nm, while a less ablation time (5 minutes) produces a lower concentration of Au NPs and average particle size d_50_ of 401 ± 33 nm. However, the disadvantage of a longer ablation time is that it produces trimodal size distribution peaks of 2 nm, 25 nm, and 131 nm.

**Fig. 2:**
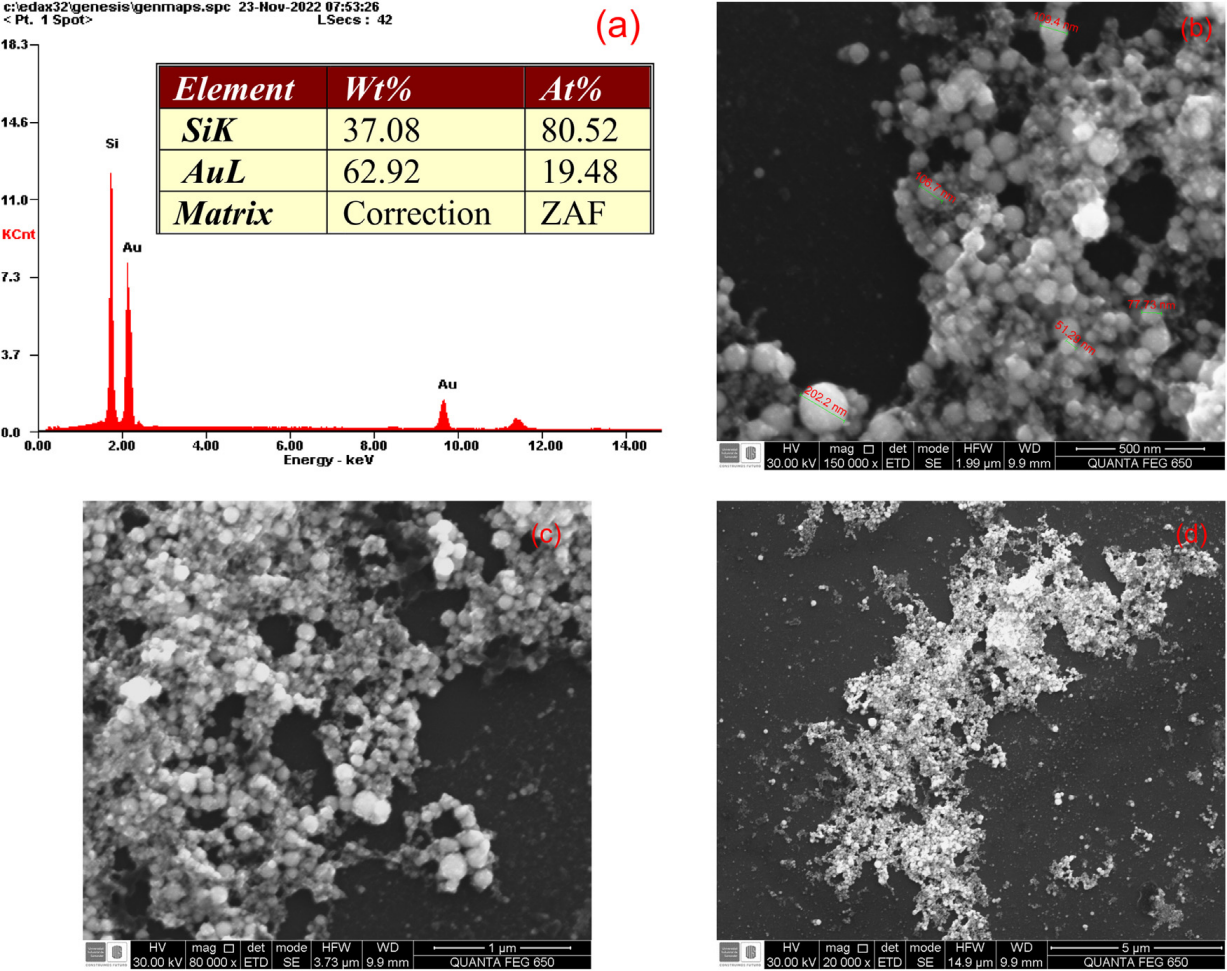
(a) The weight percentage of elements from EDS analysis and SEM images of Au NPs dried on a silicon blank with magnifications of (b) 500 nm, (c) 1 μm, and (d) 5 μm.

On the other hand, Au NPs were observed with a scanning electron microscope (SEM) to provide their morphology and establish a comparison with the average particle size from DLS measurements. SEM images were obtained with the equipment FEI QUANTA FEG 650 SEM. Besides, this equipment is configured with an acceleration voltage of 25 kV, and it has detectors: Everhart Thornley ETD for secondary electrons (SE), and backscattered electron (BSE) type SDD EDAX APOLO X for chemical analysis of energy dispersive spectroscopy (EDS), which allows determining the chemical composition of a material with a resolution of 126.1 eV (Mn Kα). The EDX Genesis software processed the data from EDS measurements to provide semi-quantitative information about the chemical elements on a prepared Au NPs sample, as is shown in Fig. 2(a). Therefore, the Au NPs were dropped on a silicon blank oriented at (110) crystal plane and dried on a heating plate at 60°C until reaching a thin film to be observed in SEM images. Then, Fig 2(a) confirms the presence of gold material at approximately 63 wt% on the scanned section in Fig. 2 (b). In addition, Fig. 2(b) with a magnification of 500 nm allows to note the spherical morphology of Au NPs and calculate their average size. This value is around 110 ± 51 nm, which is in the particle size range estimated by DLS measurements. While Figs. 2(c) and 2(d) correspond with the magnifications of 1 μm and 5 μm, respectively. Previous magnifications show how the Au NPs have agglomerated after the dried stage; thus, they surely do when incorporated into the cement matrix. Finally, the ultraviolet-visible light absorbance of the obtained particles was measured using an Agilent 8453 UV-visible spectrometer equipped with a deuterium discharge and tungsten lamps in 1 mm optical path quartz cuvettes. Herein, it was observed π-plasmon representative peak of Au NPs at 511 ± 0.0003 nm, which is another manner to confirm its presence in the water dispersion [7].

## Cement-based gold nanoparticles fabrication

Cement paste was hand mixed with a water-to-cement ratio (w/c) of 0.47. This value is an average of two water-cement ratios suggested by the standard test method for compressive strength of cylindrical concrete specimens C39/C39M [8]. Here, Au NPs were included in the water ratio, as shown in Eq. 1. Therefore, each specimen was prepared with 60 gr of cement and 28.2 mL or what is the same 28.2 g of ultrapure water + Au NPs.

(1) $$\frac{AuNp\;\left(gr\right)+H_{2}O\left(gr\right)}{Cement\;\left(gr\right)}=0.47$$

**Fig. 3:**
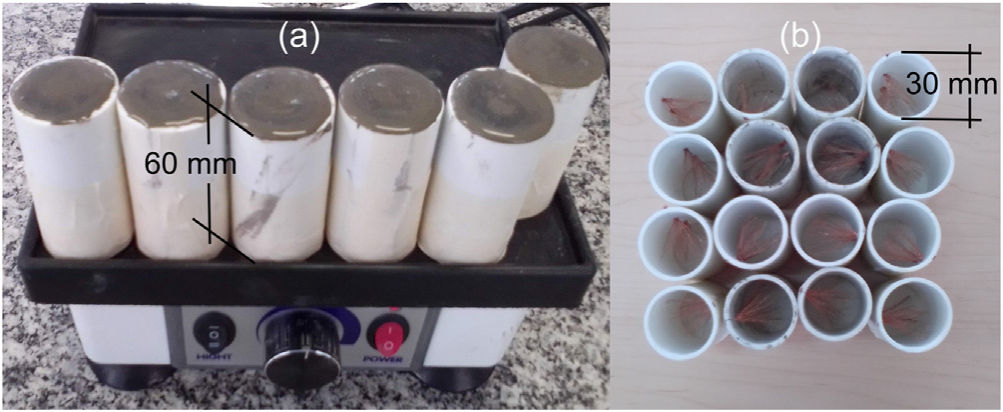
(a) Side view of cement paste into molds on a vibrating table, (b) Top view of cylindrical molds with copper wire installed. The copper wire electrodes are separated by 20 mm from each other.

Besides, specimens without Au NPs were used as a reference. More details about the material proportion used for reference, 442 ppm Au NPs, and 658 ppm Au NPs specimens are presented in Table 1. Once the cement composite mixing is obtained, the cement paste is poured of 60 mm height and 30 mm diameter Polyvinyl chloride (PVC – (C_2_H_3_Cl)n) cylindrical molds as it is used in the original method [9]. Firstly, the cement with Au NPs+H_2_O in a beaker was mixed until the mix was homogenized. It is essential to clarify that no surfactant was included in this stage to avoid a masking effect on the hydration kinetics or reactions between Au NPs and Portland cement [10], i.e., interactions that reduced the electric charge transfer between adjacent Au NPs. After, the mold was sealed with mineral oil to prevent the cement paste from sticking to the walls of the cylinder, and finally, the filling process was made with the deposition of 3 equal layers through the compaction method of damping and vibration. The molds were put on a small vibrating table for 10 min to reduce the pores induced by the hydration process in the cementitious material (see Fig. 3(a)). Besides, they were unmolded and dried at room temperature for 48 hours; later, they were cured in ultra-pure water for 28 days.

**Table 1:** The proportion of Au NPs-cement composites (w/c: water-to-cement ratio).

Material	Reference	442 ppm Au NPs	658 ppm Au NPs
Cement (g)	60.0	60.0	60.0
Water (g)	28.2	28.2	28.2
Au Nanoparticles (mg)	-	0.39	0.58
w/c	0.47

The molds had two 3 mm diameter holes at 1/3 and 2/3 of the total height; these holes were used to embed the copper wire into each sample to work as measuring electrodes. Desoldering copper wire ref. ZD-180 had a width of 3 mm, and it was made of a wire's net of 0.05 mm that was extended as a plane inside the cylindrical mold. Configuration of the molds and positioning of the copper electrodes is presented in Fig. 3(b).

**Fig. 4:**
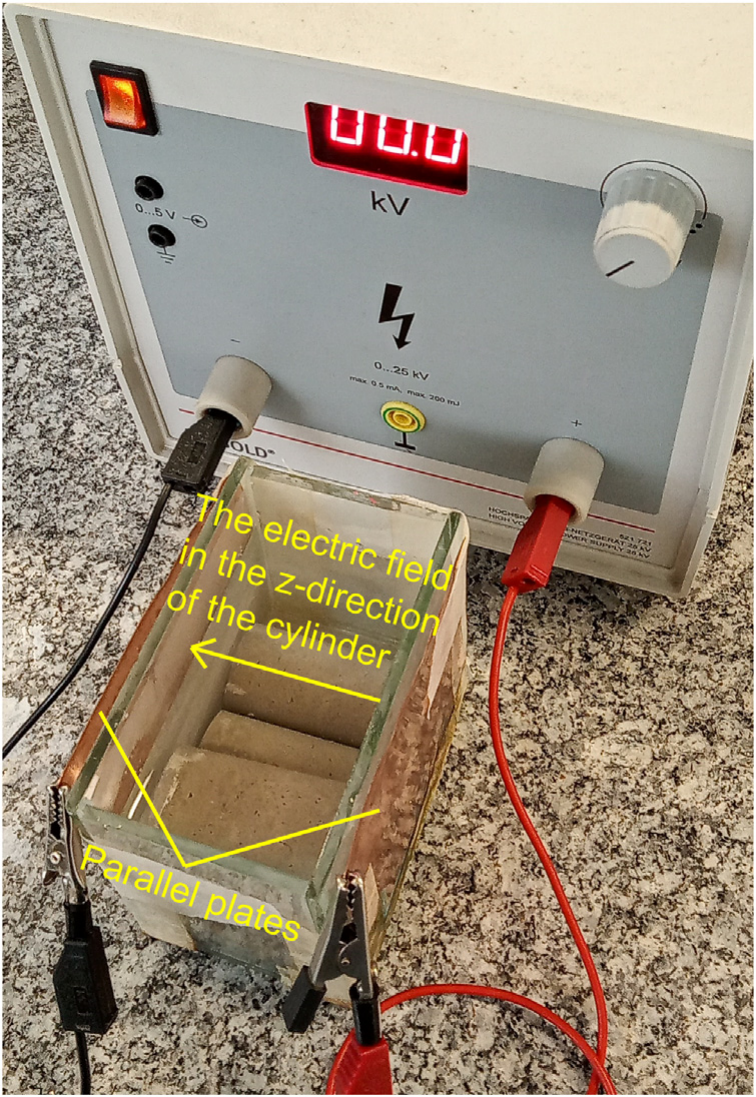
Curing stage of Au NPs-cement composite under an external electric field in the axial direction.

A group of specimens with different Au NPs concentrations were cured and subjected to an external electric field ($\vec{E}$) to increase the cement paste's inherent piezoelectric response [11,12]. The electric field was produced by two copper plane electrodes of sides 13 cm and 10 cm, which were oriented to pointing $\vec{E}$ in the axial direction of the cylinders, using a DC source at 20.5 V throughout the curing time (28 days). This curing method is presented in Fig. 4. After the curing stage, the specimens cured and not cured under an electrical field were placed into a furnace at 40^°^C for 24 hours. Next, the specimens were denoted with the lowercase letter (p) followed by the fabrication sequence number, for instance, p10. In that sense, Table 2 is shown the nomenclature of Au NPs-cement composites.

**Table 2:** Nomenclature of Au NPs-cement composites studied with this method.

Specimens	Curing	Curing + Electric Field
Reference	p3, p4, p5, p6	p1, p2, p7
442 ppm Au NPs	p8, p9, p10	p11, p12, p13
658 ppm Au NPs	p14, p15	p16, p17

## Electric (AC) and piezoelectric characterizations

**Fig. 5:**
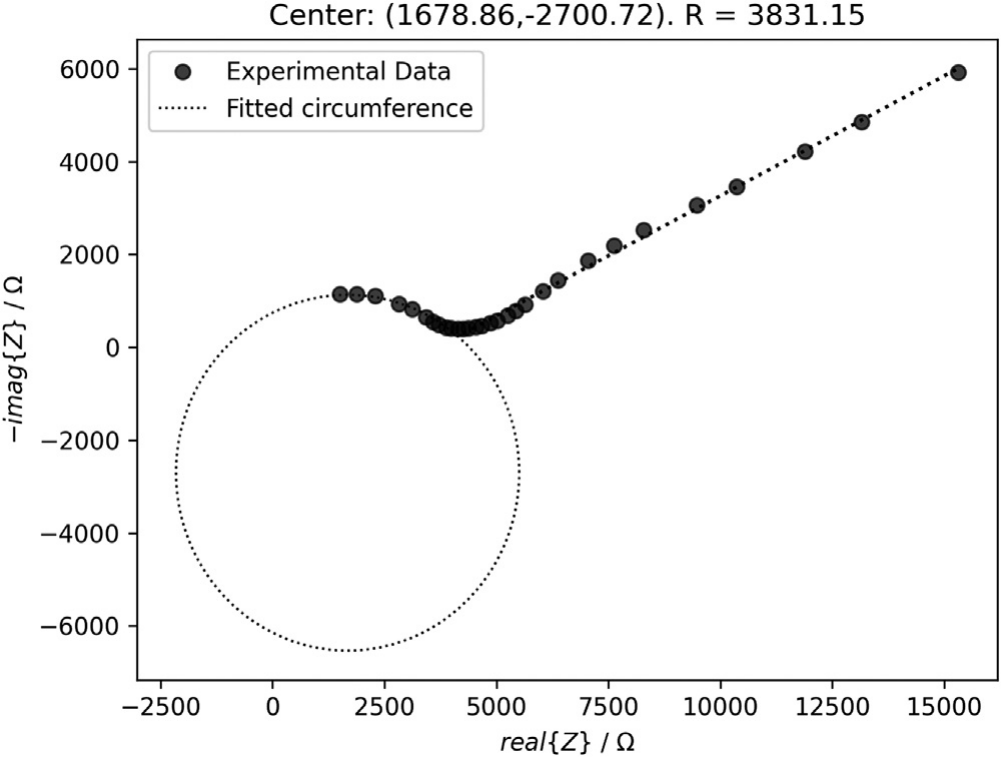
Nyquist plot of a reference specimen and its fitted semicircle.

The electrical characterization in alternating current (AC) was performed through electrical impedance spectroscopy, which was carried out by setting an AC potential signal of 10 mV while the frequency was swept from 1 MHz to 100 mHz in 60 evenly distributed points. The polarization resistance was found by fitting Nyquist's plots from a geometrical decomposition divided by the semicircle at high and middle frequencies and the slope at or constant phase element (CPE) at low frequency. In this light, a Python script called “semiCirclesEISModel()” was developed with programming methods to search the polarization resistance and the CPE. The method “search_min_max()” consists of a searcher of minimum and maximum values of a function in a list of 60 elements. In addition to the function's divergence, the searcher method considers the product of the actual point with two following points of the divergence to detect slope changes in the function and error points induced by electromagnetic noise or measurement errors. With previous information, another programming method gets the semicircles of Nyquist's plots to find the intercept on the real impedance axis (the polarization resistance). An example to show the capabilities of the Python script is shown in Fig. 5.

**Fig. 6:**
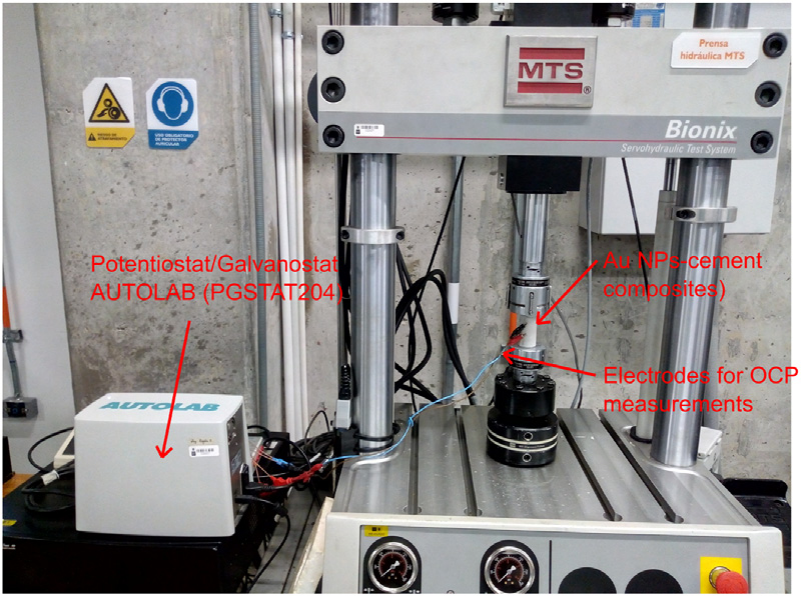
Electromechanical setup to get the piezoelectric properties of the samples.

Regarding the piezoelectric response, the samples were placed in a universal testing machine (model MTS-Bionix, maximum force capacity 25 kN). In this equipment, a compressive strength until 2.0 kN was applied in the axial direction at a constant loading rate of 0.02 kN/s, and simultaneously, the open-circuit potential (OCP) was also measured, i.e., the voltage produced by the cement sensor when an axial force compresses it. It should be noted that no external voltage was applied during the OCP measurements to guarantee that all results corresponded to piezoelectric effects. Besides, data from the universal testing machine and the potentiostat were extracted as .txt files. The experimental setup used is presented in Fig. 6. Both electrical impedance and OCP were measured using a potentiostat/galvanostat AUTOLAB model PGSTAT204. It uses Nova 2.1 software as graphic interphase to select and control the measurement procedure. The extracted files from OCP measurements were processed by a second Python script named “pro_data()”, herein were used mainly the methods “get_ocp()” and “get_force()” to combine data from mechanical and electrical measurements.

Linear regression was used to establish the effect of the compressive force on the generated voltage. Both measurements were acquired in the time domain. It is known that there is a linear relationship between electrical output and mechanical input based on the constitutive equations of piezoelectricity [13]. Hence, the compressive force and the electric polarization inside the sample change in the same time interval, and the force rate is slower than the internal polarization changes (it does not consider a delay between both quantities). Then, the generated voltage and force series were interpolated using the Phyton script, and time was used to equalize the generated voltage with the compressive force, as is shown in Eq. 2. It was necessary to interpolate the time measurements in both OCP and mechanical through a third programming method called “plot_ocp_force()” in the “pro_data()” module due to the time intervals of the two machines were different.

(2) V=γF+β

**Fig. 7:**
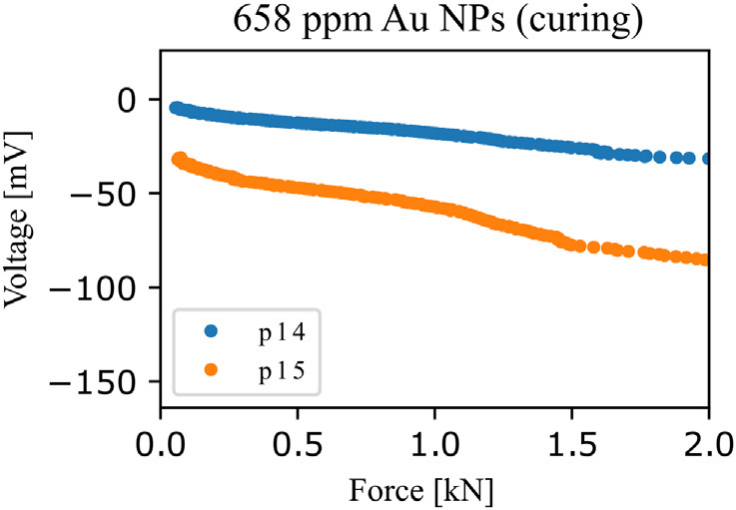
Generated voltage-Force curves from the specimens with Au NPs concentrating at 658 ppm.

In Eq. 2, V is the OCP (V), F is the applied force (kN), β is the initial OCP (i.e., at zero force), and γ is the OCP-mechanical coefficient mV/kN. The OCP-mechanical coefficient is defined as $\gamma =\frac{m_{v}}{m_{F}}$, where  $m_{v}$ is the measured OCP rate and $m_{F}$ is the applied force rate. The constant γ can be multiplied by the factor A/L (A is the cross-section, and L is the distance between electrodes) to obtain the piezoelectric voltage parameter g_33_ (generally presented in units of $10^{-5}$mVm/N), as demonstrated Al-Qaralleh [14] for blended cement pastes cured under an external electric field and validated going forward by Triana-Camacho et al. for graphene oxide-cement composites [12]. For instance, in Fig. 7, we show the results of the measure-software methods. Herein, the piezoelectric response of Au NPs-cement composites (p14 and p15) increases until 72.35 ± 24.31 $10^{-5}$mVm/N. In comparison with the reference specimens with a piezoelectric voltage parameter of 2.54 ± 1.91 $10^{-5}$mVm/N, the piezoelectric response has been improved approximately 28 times by incorporating Au NPs into the cement matrix.

## CRediT authorship contribution statement

**Daniel A. Triana-Camacho:** Methodology, Software, Formal analysis, Writing – original draft, Writing – review & editing, Conceptualization, Validation. **Rogelio Ospina-Ospina:** Writing – review & editing, Resources, Visualization. **Jorge H. Quintero-Orozco:** Supervision, Funding acquisition, Resources, Validation, Writing – review & editing.

## Declaration of Competing Interest

The authors declare that they have no known competing financial interests or personal relationships that could have appeared to influence the work reported in this paper.

## Data Availability

Data will be made available on request.
